# The Significance of Splenectomy for Advanced Proximal Gastric Cancer

**DOI:** 10.1155/2012/301530

**Published:** 2012-05-21

**Authors:** Atsushi Nashimoto, Hiroshi Yabusaki, Atsushi Matsuki

**Affiliations:** Department of Surgery, Niigata Cancer Center Hospital, Niigata 951-8566, Japan

## Abstract

*Objectives*. The significance of splenectomy in advanced proximal gastric cancer is examined retrospectively. *Methods.* From 1994 to 2004, 505 patients with advanced proximal gastric cancer underwent curative total gastrectomy with preserving spleen (T) for 264 patients and total gastrectomy with splenectomy (ST) for 241 patients. *Results*. Patients who underwent splenectomy showed more advanced lesions. The metastatic rate of lymph node (LN) in the splenic hilus (No. 10) in ST was 18.3%. As for the incidence of surgical complications, there was not statistically difference except for pancreatic fistula. The index of estimated benefit of (No. 10) LN was 4.2, which was similar to that of (No. 9), (No. 11p), (No. 11d), and (No. 16) LNs. 5-year survival rate of (No. 10) positive group was 22.2%. 5-year survival rates of pSE and pN2 in T group were better than that of pSE and pN2 in ST, respectively. The superiority of ST was not confirmed even in Stage II, IIIA, and IIIB. *Conclusion*. Splenectomy was not effective for patients with (No. 10) metastasis in long-term survival. Spleen-preserving total gastrectomy will be feasible and be enough to accomplish radical surgery for locally advanced proximal gastric cancer.

## 1. Introduction

Although it is well known that lymph node (LN) metastasis is an important factor in the prognosis of gastric cancer, the optical extent of LN dissection remains controversial. Splenectomy has been indicated to remove the LNs surrounding the splenic artery (No. 11) and splenic hilum (No. 10). Previous reports suggested that gastrectomy with splenectomy resulted in better survival than gastrectomy alone in gastric cancer patients [1]. The Japanese retrospective studies revealed that the frequency of LN metastasis to No. 10 in proximal gastric cancer was 15–20%, and the 5-year survival rate was 20–25% [2, 3]. Total gastrectomy with splenectomy is considered to be a standard procedure for proximal advanced gastric cancer in gastric cancer treatment guidelines [4]. But two large prospective randomized trials in western countries reported that splenectomy was a risk factor for morbidity and mortality [5, 6]. Preservation of the spleen during extended lymphadenectomy decreases complications with no clear evidence of improvement or detriment to overall survival [7]. Then modified D2 lymphadenectomy avoiding splenectomy is now accepted as a standard procedure in the west countries. Our retrospective study was designed to investigate the significance of splenectomy by evaluating postoperative morbidity, frequency of the each LN metastasis, and long-term surgical outcomes of locally advanced proximal gastric cancer patients who underwent total gastrectomy with R0 resection.

## 2. Patients and Methods

### 2.1. Pathological Examination of Lymph Nodes

**Figure 1:**
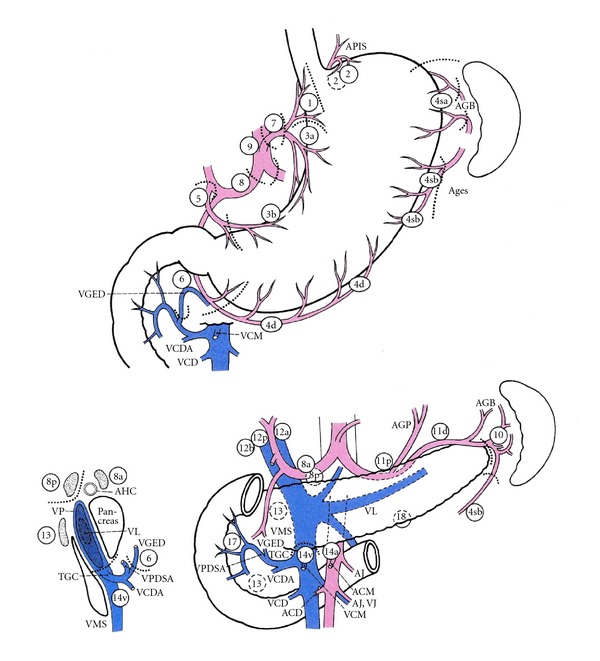
Lymph noses (LNs) are retrieved from the en bloc resected specimen and placed on the map exactly as they were in situ and numbered. Regional lymph node stations are defined as No. 1, right paracardial LN; No. 2, left paracardial; No. 3, LN along the lesser curvature; No. 4sa, LN along the short gastric vessels; No. 4sb, LN along the left gastroepiploic vessels; No. 4d, LN along the right gastroepiploic vessels; No. 5, suprapyloric LN; No. 6, infrapyloric LN; No. 7, LN along the left gastric artery; No. 8a LN along the common hepatic artery; No. 9, LN around the celiac artery; No. 10 LN at the splenic hilum; No. 11p, LN along the proximal splenic artery; No. 11d, LN along the distal splenic artery; No. 12a, LN in the hepatoduodenal ligament; No. 13, LN on the posterior surface of the pancreatic head; No. 14v, LN along the superior mesenteric vein; No. 16, LN around the abdominal aorta. APIS, left inferior phrenic artery; GB, short gastric artery; AGES, left gastroepiploic artery; VCM, middle colic vein; VGED, right gastroepiploic vein; VCDA, accessory right colic vein; VCD, right colic vein; AGP, posterior gastric artery; VL, splenic vein; AJ, jejunal artery; VJ, jejunal vein; ACM, middle colic artery; ACD, right colic artery; TGC, gastrocolic trunk; VMS, superior mesenteric vein; VPDIA, anterior inferior pancreaticoduodenal vein; AHC, common hepatic artery; VP, portal vein.

All regional LNs were separated immediately after gastrectomy by the operators. Node numbers were recorded using a LN map (Figure 1). Nodes were assigned to the appropriate anatomical stations according to Japanese Classification of Gastric Carcinoma (JCGC) of the 2nd English edition [8]. Nodes found at each station were labeled and immediately sent for histological examination.

### 2.2. Patient Population

From 1994 to 2004, 505 patients with a single gastric adenocarcinoma located in the upper third portion underwent curative total gastrectomy at Niigata Cancer Center Hospital. Among them, 240 patients underwent total gastrectomy with splenectomy (ST), because the tumor involved the greater curvature or enlarged LN of No. 10 and/or No. 11. The remaining 265 patients underwent spleen-preserving lymphadenectomy (T) and remove No. 11 but not No. 10. The clinicopathological features, stage and 5-year survival rates according to JCGC were compared between ST group and T group.

### 2.3. Procedures

Total gastrectomy with D2 and more extensive lymphadenectomy was performed according to the rules of the JCGC. The standard reconstruction was Roux-en Y method. In T group, No. 11 was dissected along the upper border of the pancreas but not No. 10 with or without mobilization of the spleen from the retroperitoneum. When the tumor involved the greater curvature and/or enlarged LN suspected metastasis at splenic hilum was found before or during operation, splenectomy was performed simultaneously as R0 resection. The index of estimated benefit from lymphadenectomy was calculated by multiplying the incidence of each nodal station by the 5-yer survival rate of patients with metastasis to that nodal station [9].

### 2.4. Statistical Analysis

All statistical analyses were conducted using the statistical program SPSS version 19 for Windows (SPSS, Chicago, IL, USA). Clinicopathological variables were analyzed using the chi-square test and the Student's *t*-test. The risk factors for No. 10 metastasis were determined using logistic regression analysis. Cumulative survival rates were calculated by the Kaplan-Meier method, and the significance of the differences in survival was determined by the log-rank test. *P*-value of <0.05 was considered statistically significant. 

## 3. Results

### 3.1. Comparison of the Clinicopathological Features

Clinicopathological features are shown in Table 1. There was no statistical difference in age and gender between ST group and T group. But there were significant differences between two groups regarding gross type, tumor location, and histological type, depth of the tumor invasion, and status of lymph node metastasis. Namely, type 3 and type 4, UML (U; the upper, M; the middle, L; the lower), undifferentiated type, pT3 and pT4, and pN2 and pN3, are found frequently in ST group. Patients who underwent splenectomy showed more advanced lesions.

### 3.2. Perioperative Morbidity

Postoperative complications were listed in Table 2. There was no significant difference between two groups concerning nonsurgical complications. The incidence of surgical complications regarding anastomotic leakage, pancreatic fistula, postoperative ileus, and intra-abdominal bleeding was higher in ST group than in T group. But there was no statistical difference except for pancreatic fistula (*P* = 0.008).

### 3.3. Lymph Node Metastasis in ST Group

**Figure 2:**
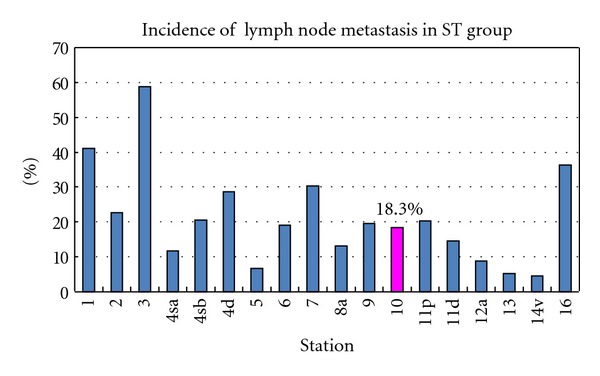
Incidence of each lymph node metastasis in ST group. The metastatic rate of the splenic hilar LN (No. 10) was 18.3%.

The lymph node metastatic rate in ST group was shown in Figure 2. No. 3 metastatic rate was highest (58.8%). The incidence of No. 10 metastasis was 18.3%, which was similar to that of No. 4sb (20.5%), No. 6 (19%), No. 9 (19.5%), and No. 11p (20.2%). No. 16 metastatic rate was 36.3% which was unexpectedly high.

**Figure 3:**
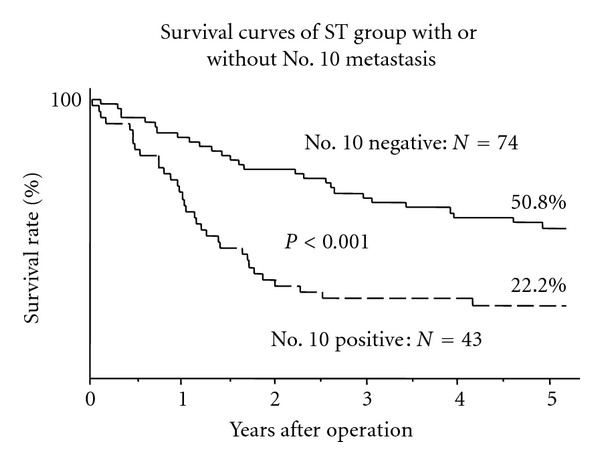
Comparison of cumulative survival curves of ST group between with or without No. 10 metastasis. The prognosis of the patients with No. 10 positive was poorer than that of the patients with No. 10 negative (*P* < 0.001).

The 5-year survival rate was 22.2% in patients with No. 10 metastasis and 50.8% in patients without its metastasis in ST group (Figure 3).

### 3.4. The Therapeutic Value of Lymph Node Dissection

**Figure 4:**
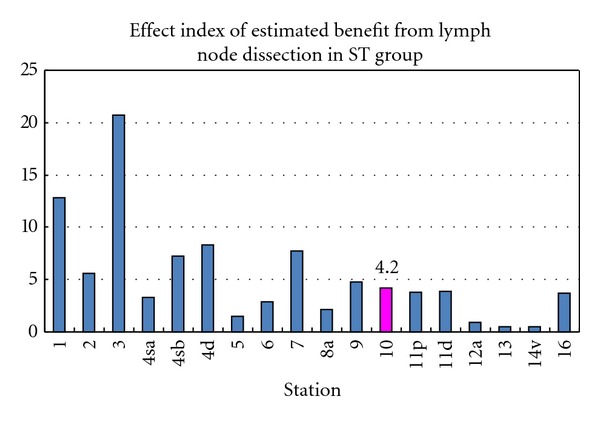
Effect index of estimated benefit from lymphadenectomy in ST group. The index was calculated by multiplication of the frequency of metastasis to the station and the 5-year survival rate of patients with metastasis to that station. The index of estimated benefit of No. 10 was approximately equal to that of No. 9, No. 11p, No. 11d, and No. 16.

The therapeutic value of extended lymph node dissection was estimated by multiplication of incidence of lymph node metastasis and 5-year survival rate of patients with metastasis for each station. The index of estimated benefit of No. 10 was 4.2, which was similar to that of No. 9 (4.8), No. 11p (3.8), No. 11d (3.9), and No. 16 (3.7) (Figure 4). Almost all the regional lymph nodes of upper third portion of the stomach had high effect index of lymphadenectomy, but the treatment index of No. 4a and No. 8a was lower than that of No. 10. 

### 3.5. Survival

**Figure 5:**
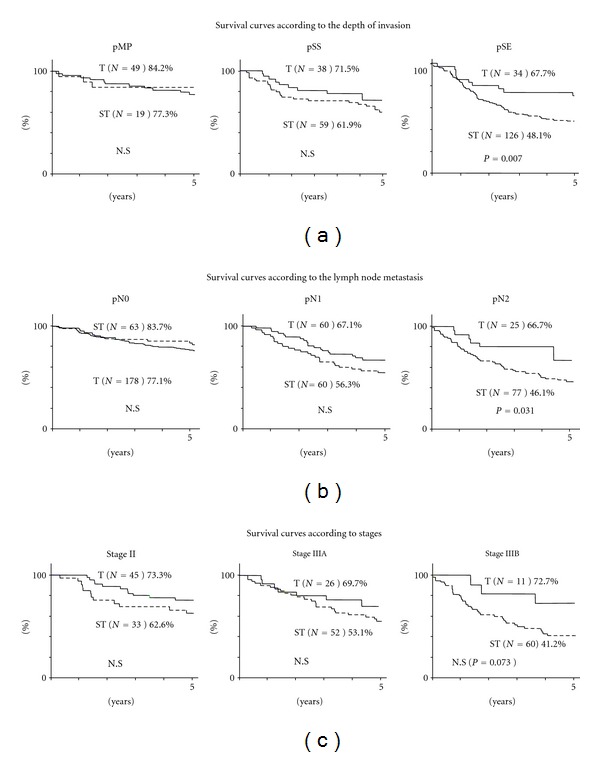
(a) Cumulative survival rates according to the depth of invasion (pT). As for pMP and pSS, there was no difference between T group and ST group, but the survival of the T group with pSE was better than that of ST group with pSE (*P* = 0.007). (b) Cumulative survival rates according to lymph node metastasis (pN). There was no difference in the cumulative survival rates between two groups with pN0 and pN1, but the survival of T group with pN2 was better than that of ST group with pN2 (*P* = 0.031). (c) Cumulative survival curves according to stage (pStage). There was no significant difference in the cumulative survival rates between two groups with Stage II, Stage IIIA, and Stage IIIB.

In the survival rate according to depth of tumor invasion, ST group revealed lower prognosis compared with T group, but there was no significant difference between two groups in T2a and T2b (Figure 5(a)). 

But the survival rate for patients with pSE (T3: tumor penetration of serosa), there was significantly difference between ST group (48.1%) and T group (67.7%). In the survival rate according to lymph node metastasis, there was no significant difference in the cumulative survival rates between two groups in pN0 and pN1 (Figure 5(b)). 

But in the survival rate for patients with pN2, there was significantly difference between ST group (46.1%) and T group (66.7%). As for the survival rate according to stage, the survival of ST group was lower than that of T group in stages II, IIIA, and IIIB, but there was no significant difference (Figure 5(c)).

## 4. Discussion

The current standard treatment for proximal advanced gastric cancer in Japan is total gastrectomy with D2 lymphadenectomy. In order to accomplish D2 lymphadenectomy, splenectomy had been justified for complete removal of No. 10 as extended radical surgery. But extended resection which is regarded as a standard procedure in Asian countries is not effective in Western countries. The splenectomy caused high morbidity and mortality, and it was shown to be an independent prognostic risk factor on multivariate analysis in node-negative patients in previous studies [10–15]. On the other hand, the splenectomy is considered to be a safe procedure that does not decrease surgical mortality [16]. A Korean trial has also reported that postoperative morbidity after splenectomy for D2 lymphadenectomy was not higher than simple total gastrectomy, but there was no significant difference in 5-year survival between with and without splenectomy [17]. Patients with proximal advanced gastric cancer localized on the greater curvature and type 4 might obtain relatively high survival benefits from No. 10 lymphadenectomy [18]. The splenectomy has become a safe technical procedure, but the surgical procedure of a total gastrectomy with splenectomy should be performed at a high volume hospital to avoid the postoperative complications.

The frequency of No. 10 metastasis was reported to be high in proximal advanced gastric cancer located on the greater curvature or in the posterior wall of the stomach, and lymphatic pathways along the posterior gastric artery, splenic artery, short gastric vessels, and/or gastroepiploic vessels were suggested to be important for No. 10 metastasis [19]. Lymphography has demonstrated that the lymphatic flow from the left upper region of the stomach enters the lymph node in the splenic hilum and travels to the nodes around the celiac trunk along the splenic artery [20]. In our study, the location involving the greater curvature, pN3 and No. 11d metastasis were risk factors for No. 10 metastasis, and the frequency of No. 10 metastasis was similar to that of No. 4sb, No. 9, and No. 11p metastasis. Furthermore, LN dissection effect index of No. 10 was almost as same as that of No. 9, No. 11p, and No. 11d. But the prognosis of patients with No. 10 metastasis was still poor even after its dissection. Furthermore, splenectomy does not improve survival of patients with proximal advanced gastric cancer even though curative resection was performed [21, 22]. Multivariate analysis demonstrated that nodal metastasis was independent prognostic factor, but splenectomy was not [23]. These reports suggested that the patients with No. 10 metastasis had already too extended LN metastasis to improve the prognosis. Accordingly, the splenectomy for D2 lymphadenectomy may be unnecessary in all the patients with advanced gastric cancer. On the contrary, some authors have found the survival benefit and recommended splenectomy for No. 10 lymphadenectomy. The splenectomy was one of the independent prognostic factors, and total gastrectomy with splenectomy is recommended for patients with No. 10 positive T3 proximal gastric cancer [24]. The survival of No. 10 positive patients was not to be different from that of No. 10 negative patients when curative surgery was performed [25]. The splenectomy was recommended when the tumor was located on the greater curvature or posterior wall of the stomach and had No. 4sa, No. 4sb, or No. 11 metastasis [19]. In fact, it is difficult to detect the depth of tumor invasion and No. 10 and/or No. 11 metastasis though a preoperative and intraoperative diagnostic technique. In Germany, No. 10 metastasis was observed only in advanced cancer, particularly in tumors located in the greater curvature and/or type 4 tumors [26]. Our current study showed that splenectomy adversely affected survival in pSE and pN2, while there was no significant difference in survival rates in pMP, pSS, pN0, and pN1 and among Stage II, IIIA, and IIIB. Though there were limitations of our study which was retrospectively conducted in a single institute, and there was selection bias, our study would suggest the benefit of spleen preservation on postoperative morbidity and long-term surgical outcomes. The overall survival rate stratified by stage was analyzed in a prospective randomized controlled trial [27], in which the 5-year overall survival rates of patients with stageI, stage II, and stage III were not significantly different between the 2 groups. Until 2005, our institute preferred to perform a total gastrectomy with splenectomy in advanced proximal gastric cancer for complete D2 lymphadenectomy. Recently we had a policy of splenectomy for the patients with No. 10 enlargement in the splenic hilum suggesting metastasis or tumor located in greater curvature or encircling in upper third portion of the stomach. A randomized controlled trail to evaluate total gastrectomy with splenectomy for proximal advanced gastric carcinoma with R0 resection (JCOG0110-MF) [28] has already recruited 505 patients and resulted that splenectomy was associated with higher morbidity and larger blood loss and was safely performed by specialized surgeons with low mortality. The precise impact of splenectomy on prognosis remains uncertain and the impact on long-term survival should be awaited. 

In conclusion, although splenectomy for patients with proximal advanced gastric cancer was not an important risk factor for postoperative morbidity, splenectomy was not effective for patients with No. 10 metastasis in long-term survival. Spleen-preserving total gastrectomy will be feasible and be enough to accomplish radical surgery for locally advanced proximal gastric cancer.

## Figures and Tables

**Table 1:** Clinicopathological characteristics of the patients who underwent total gastrectomy with or without splenectomy (*N* = 505).

Characteristics	T *N* = 265 (%)	ST *N* = 240 (%)	*P* value
Age (year)			0.121
<70	163 (61.5)	18 (75.0)	
≥70	102 (39.5)	60 (25.0)	
Age (year)			0.481
Male	198 (74.7)	172 (71.7)	
Female	67 (25.3)	68 (28.3)	
Gross type			<0.001
Type 0, 1, 2	221 (83.4)	100 (41.7)	
Type 3, 4	44 (16.6)	140 (58.3)	
Tumor location			<0.001
U	191 (72.1)	159 (66.3)	
M, L	60 (22.6)	34 (14.2)	
UML	14 (5.3)	47 (19.6)	
Histological type			<0.001
Differentiated	151 (57.0)	97 (40.4)	
Undifferentiated	114 (43.0)	143 (59.6)	
Depth of invasion			<0.001
pT1, T2	228 (86.0)	78 (32.5)	
pT3, T4	37 (14.0)	162 (67.5)	
Lymph node metastasis			<0.001
pN0, N1	220 (83.0)	123 (51.3)	
pN2, N3	45 (17.0)	(48.8)	

*U; upper third, M; middle third, L; lower third.

**Table 2:** Perioperative morbidity following total gastrectomy with or without splenectomy.

Complication	T (without splenectomy)	ST (with splenectomy)	*P* value
Nonsurgical complication			
Cardiovascular	3 (1.1)	2 (0.8)	N.S.
Pulmonary	7 (2.6)	8 (3.3)	N.S.
Liver dysfunction	0	2 (0.8)	N.S.
Renal dysfunction	0	2 (0.8)	N.S.
CNS disorder	2 (0.8)	2 (0.8)	N.S.
Others	6 (2.3)	7 (2.9)	N.S.
Surgical complication			
Anastomotic leakage	1 (0.4)	4 (1.6)	N.S.
Panctreatic fistula	16 (6.0)	31 (12.9)	0.008*
Postoperative	22 (8.3)	21 (8.7)	N.S.
Bleeding	0	3 (1.3)	N.S.

N.S., not significant. *significant difference.

## References

[1] Toge T, Kameda A, Kuroi K, Seto Y, Yamada H, Hattori T (1985). The role of the spleen in immunosuppression and the effects of splenectomy on prognosis in gastric cancer patients. *Nippon Geka Gakkai zasshi*.

[2] Okajima K, Isozaki H (1995). Splenectomy for treatment of gastric cancer: Japanese experience. *World Journal of Surgery*.

[3] Ishikawa S, Shimada S, Miyanari N, Hirota M, Takamori H, Baba H (2009). Pattern of lymph node involvement in proximal gastric cancer. *World Journal of Surgery*.

[4] Sano T, Aiko T (2011). Japanese gastric cancer treatment guidelines 2010 (ver. 3). *Gastric Cancer*.

[5] Bonenkamp JJ, Hermans J, Sasako M, Van De Velde CJH (1999). Extended lymph-node dissection for gastric cancer. *New England Journal of Medicine*.

[6] Cuschieri A, Fayers P, Fielding J (1996). Postoperative morbidity and mortality after D1 and D2 resections for gastric cancer: preliminary results of the MRC randomised controlled surgical trial. *Lancet*.

[7] Brar SS, Seevaratnam R, Cardoso R A systematic review of spleen and pancreas preservation in extended lymphadenectomy for gastric cancer.

[8] Gastric Cancer Association Japanese (1998). Japanese classification of gastric carcinoma: 2nd English edition. *Gastric Cancer*.

[9] Sasako M, McCullouch P, Kinoshita T, Maruyama K (1995). New method to evaluate the therapeutic value of lymph node dissection for gastric cancer. *British Journal of Surgery*.

[10] Brady MS, Rogatko A, Dent LL, Shiu MH (1991). Effect of splenectomy on morbidity and survival following curative gastrectomy for carcinoma. *Archives of Surgery*.

[11] Griffith JP, Sue-Ling HM, Martin I (1995). Preservation of the spleen improves survival after radical surgery for gastric cancer. *Gut*.

[12] Kwon SJ, Lee JI, Choi DW (1997). Prognostic impact of splenectomy on gastric cancer: results of the Korean Gastric Cancer Study Group. *World Journal of Surgery*.

[13] Wanebo HJ, Kennedy BJ, Winchester DP, Stewart AK, Fremgen AM (1997). Role of splenectomy in gastric cancer surgery: adverse effect of elective splenectomy on longterm survival. *Journal of the American College of Surgeons*.

[14] Maehara Y, Moriguchi S, Yoshida M, Takahashi I, Korenaga D, Sugimachi K (1991). Splenectomy does not correlate with length of survival in patients undergoing curative total gastrectomy for gastric carcinoma: univariate and multivariate analyses. *Cancer*.

[15] Otsuji E, Yamaguchi T, Sawai K, Okamoto K, Takahashi T (1999). Total gastrectomy with simultaneous pancreaticosplenectomy or splenectomy in patients with advanced gastric carcinoma. *British Journal of Cancer*.

[16] Tanizawa Y, Terashima M (2010). Lymph node dissection in the resection of gastric cancer: review of existing evidence. *Gastric Cancer*.

[17] Yu W, Choi GS, Chung HY (2006). Randomized clinical trial of splenectomy versus splenic preservation in patients with proximal gastric cancer. *British Journal of Surgery*.

[18] Kosuga T, Ichikawa D, Okamoto K (2011). Survival benefits from splenic hilar lymph node dissection by splenectomy in gastric cancer patients: relative comparison of the benefits in subgroups of patients. *Gastric Cancer*.

[19] Aoyagi K, Kouhuji K, Miyagi M (2010). Prognosis of metastatic splenic hilum lymph node in patients with gastric cancer after total gastrectomy and splenectomy. *World Journal of Hepatology*.

[20] Maruyama K, Okabayashi K, Kinoshita T (1987). Progress in gastric cancer surgery in Japan and its limits of radicality. *World Journal of Surgery*.

[21] Zhang CH, Zhan WH, He YL, Chen CQ, Huang MJ, Cai SR (2007). Spleen preservation in radical surgery for gastric cardia cancer. *Annals of Surgical Oncology*.

[22] Shin SH, Jung H, Choi SH (2009). Clinical significance of splenic Hilar lymph node metastasis in proximal gastric cancer. *Annals of Surgical Oncology*.

[23] Lee KY, Noh SH, Hyung WJ (2001). Impact of splenectomy for lymph node dissection on long-term surgical outcome in gastric cancer. *Annals of Surgical Oncology*.

[24] Huang CM, Wang JB, Lu HS (2009). Prognostic impact of splenectomy on advanced proximal gastric cancer with No. 10 lymph node metastasis. *Chinese Journal of Surgery*.

[25] Ikeguchi M, Kaibara N (2004). Lymph node metastasis at the splenic hilum in proximal gastric cancer. *American Surgeon*.

[26] Monig SP, Collet PH, Baldus SE (2001). Splenectomy in proximal gastric cancer: frequency of lymph node metastasis to the splenic hilus. *Journal of Surgical Oncology*.

[27] Csendes A, Burdiles P, Rojas J, Braghetto I, Diaz JC, Maluenda F (2002). A prospective randomized study comparing D2 total gastrectomy versus D2 total gastrectomy plus splenectomy in 187 patients with gastric carcinoma. *Surgery*.

[28] Sano T, Yamamoto S, Sasako M (2002). Randomized controlled trial to evaluate splenectomy in total gastrectomy for proximal gastric carcinoma: Japan clinical oncology group study JCOG 0110-MF. *Japanese Journal of Clinical Oncology*.

